# Impacts of COVID-19 at the intersection of substance use disorder treatment and criminal justice systems: findings from three states

**DOI:** 10.1186/s40352-022-00184-8

**Published:** 2022-08-04

**Authors:** Allyson L. Dir, Martha Tillson, Matthew C. Aalsma, Michele Staton, Monte Staton, Dennis Watson

**Affiliations:** 1 Department of Psychiatry, Indiana University School of Medicine, 410 W 10th Street, Indianapolis, IN 46202 USA; 2 Adolescent Behavioral Health Research Program, Department of Pediatrics, Indiana University School of Medicine, 410 W 10th Street, Indianapolis, IN 46202 USA; 3 Center on Drug and Alcohol Research, University of Kentucky, 643 Maxwelton Ct., Lexington, KY 40508 USA; 4 Department of Pediatrics, Section of Adolescent Medicine, Indiana University School of Medicine, 410 W 10th Street, Indianapolis, IN 46202 USA; 5 Department of Behavioral Science, College of Medicine, University of Kentucky, 117 Medical Behavioral Science Building, Lexington, KY 40504 USA; 6 Center for Dissemination and Implementation Science, Department of Medicine, University of Illinois College of Medicine at Chicago, 818 S Wolcott Ave, Chicago, IL 60613 USA; 7 Chestnut Health Systems, 221 W. Walton St., Chicago, IL 60610 USA

**Keywords:** COVID-19, Decarceration, Opioid use disorder, Substance use disorder, Telehealth, Telejustice

## Abstract

**Background:**

Individuals with substance use disorders (SUD), particularly opioid use disorder (OUD), who are criminal justice-involved are a particularly vulnerable population that has been adversely affected by COVID-19 due to impacts of the pandemic on both the criminal justice and treatment systems. The manuscript presents qualitative data and findings exploring issues related to SUD/OUD treatment among individuals involved in the justice system and the impacts of COVID-19 on these service systems. Qualitative data were collected separately by teams from three different research hubs/sites in Illinois, Indiana, and Kentucky; at each hub, data were collected from justice system personnel (*n* = 17) and community-level SUD/OUD providers (*n* = 21). Codes from two hubs were reviewed and merged to develop the cross-hub coding list. The combined codes were used deductively to analyze the third hub‘s data, and higher-level themes were then developed across all the hubs’ data.

**Results:**

Themes reflected the justice and treatment systems’ responses to COVID-19, the intersection of systems and COVID-19’s impact on providing OUD treatment for such individuals, and the use of telehealth and telejustice.

**Conclusions:**

Results highlight that despite rapid adaptations made by systems during the pandemic, additional work is needed to better support individuals with OUD who are involved in the justice system. Such work can inform longer-term public health crisis planning to improve community OUD treatment access and linkage for those who are criminal justice-involved.

## Introduction

Criminal justice-involved individuals with substance use disorders (SUD), especially opioid use disorders (OUD), are a vulnerable population existing at the intersection of two systems considerably affected by the COVID-19 pandemic: the (1) criminal justice system and (2) SUD treatment system. For example, over two-thirds of adults involved in the prison system (CASA Columbia, 2010) and over one-third of youth involved in the juvenile justice system (Wasserman et al., 2010) have SUDs, with high rates of opioid use (Winkelman et al., 2018). Recent efforts have focused on improving outcomes for such individuals. For example, the National Institute on Drug Abuse established the Justice Community Innovation Opioid Network (JCOIN) to promote research to increase the quality of care for people involved in the justice system who also misuse opioids (https://heal.nih.gov/research/research-to-practice/jcoin). However, COVID-19 has created considerable challenges for advancing such efforts. The current study focuses on the impact of COVID-19 on the criminal justice and SUD treatment systems’ ability to appropriately address SUD/OUD among justice-involved individuals.

Incarcerated persons broadly have five times the prevalence of COVID-19 infection and three times the related mortality rate compared to the general population (Montoya-Barthelemy et al., 2020; Saloner et al., 2020). COVID-19-related risks might be even greater for those incarcerated individuals with SUD and particularly those with OUD. This is because people with SUD are thought to be at greater risk of COVID-19 infection and for experiencing more adverse COVID-19 outcomes (Alexander et al., 2020; Volkow, 2020; Wei & Shah 2020). In addition to the direct risks associated with COVID-19 infection, there has been an unprecedented rise in overdoses since the start of the pandemic, a significant proportion of which are attributable to opioids (Centers for Disease Control and Prevention, 2020). Potential factors underlying this trend include risky opioid use behaviors (e.g., using alone, changing dealers, increased use intensity), greater adulteration of the illicit drug supply, disrupted treatment and service access, and increased relapse risk (Nguyen & Buxton, 2021; Niles et al., 2021; Rosenbaum et al., 2021; Schlosser & Harris, 2020; Slavova et al., 2020).

Justice-involved individuals with SUD/OUD have been substantially affected by COVID-19-related changes within both the justice and SUD treatment systems. Within jail and prison walls, multiple actions have been taken to mitigate COVID-19-related risk among the general population of incarcerated individuals. Jail and prison systems have reduced admissions, released low-risk individuals early, suspended visitations, and halted transfers. There has also been a reduction of arrests at the level of law enforcement (Reinhart & Chen, 2021; National Conference of State Legislatures, 2020). At court and community supervision levels, many systems have implemented *telejustice* procedures (National Conference of State Legislatures, 2020), which are broadly defined as the use of telephone or video services for court hearings and probation or parole meetings. While some early research on the impact of decarceration has highlighted positive effects of risk mitigation on reducing the spread of COVID-19 (Reinhart & Chen, 2021), less research has examined other outcomes associated with justice-involved populations, such as implications for the high rates of individuals in need of behavioral health services. Within the broader community, new or modified regulations have made it easier to deliver telehealth services for behavioral health and to provide medications for opioid use disorder (MOUD). For example, individuals who are stable on methadone treatment can be given a 28-day take-home supply (rather than coming to the clinic daily) and individuals interested in starting buprenorphine are able to initiate treatment with a telehealth consultation rather than an in-person appointment (Davis & Samuels, 2021).

While such rapid adaptations have shown promise, research documenting their front-line processes and impacts is limited, though crucial to support maintenance of efforts to reduce barriers to access for MOUD and other forms of treatment. Existing research has also focused on adaptations made and impacts within the criminal justice settings (i.e., changes to MOUD in jails during COVID-19; Donelan et al., 2021) or within treatment systems only (Blanco, Compton, & Volkow, 2021). No research has examined the impact of such changes on those at the *intersection* of these systems. The current manuscript represents a synthesis of qualitative data collected separately by three JCOIN-funded research hubs conducting large clinical trials focused on improving access to OUD care among this population. The goal of this work was to examine the impact of COVID-19 on justice and treatment systems, system responses to COVID-19, and implications for individuals involved in these systems.

## Methods

Three independent research teams in Illinois, Indiana, and Kentucky were connected through the JCOIN COVID-19 workgroup. JCOIN’s topical workgroups are composed of members from each of its research hubs (i.e., funded research teams) and serve as a mechanism for collaboration across the network. The three JCOIN hubs of focus are conducting clinical trials that were conceptualized and funded prior to the start of the COVID-19 pandemic. Shortly after shelter-in-place orders were implemented across the United States, all three hubs separately launched qualitative sub-studies to collect contextual data to explore the impact of the pandemic on the systems in which their clinical trials are set. Such rapid qualitative approaches have been demonstrated to contribute to understandings of responses during disease outbreaks (Vindrola-Padros et al., 2020). The Illinois (Scott, Dennis, Grella, & Watson, 2021) and Kentucky  (Staton et al., 2021) trials discussed in this paper are focused on testing MOUD linkage interventions for adults returning to the community from jail, although the Kentucky trial focuses exclusively on women. The Indiana (Aalsma et al., 2021) trial focuses on the juvenile justice system, specifically improvements to (a) screening for opioid and substance use within the justice system and (b) linkage and access to treatment for youth involved in juvenile justice.

### Data collection locations and procedures

The data represent correctional systems and treatment systems across each hub. Data collection focused on identifying system-level issues, thus, key stakeholders involved in overseeing system-level operations were recruited. Each site used a different variation of convenience sampling to identify and recruit participants. Illinois‘s sample represent *n* = 2 county jails and *n* = 3 community providers that range in services provided (e.g., residential, inpatient, and outpatient). The Kentucky sample represents stakeholders from n = 3 jails housing individuals under both state- or county-level custody, *n* = 5 SUD treatment providers (offering a range of services along the care continuum, from outpatient to residential), and administrative or supervisory staff working within the state Department of Corrections. Indiana‘s sample represents *n* = 2 county-level juvenile justice systems as well as n = 2 community mental health centers providing outpatient treatment. Both urban and rural settings are represented across all 3 hubs. All data were collected through semi-structured interviews conducted over videoconference; interviews were audio recorded and later transcribed for analysis. Table 1 provides further details regarding interviews from each state.

**Table 1:** Overview of sites and data collected

Site	Data collection time	Justice system staff (n)	Jail staff (n)	MSUD/OUD provider (n)	Method of sampling	Topics addressed
Illinois	June 2020	0	2	3	Recruited from justice & service providers project partners	Challenges, responses/adaptations, and successes related to serving people who are justice-involved and have OUD
Indiana	April–September 2020	6	0	13	Recruited from list of individuals familiar with the research project supplied by juvenile justice administrators	Justice and treatment system response to the pandemic, impact on usual practices, including challenges, adaptations, &and successes to serving youth involved in juvenile justice and seeking treatment for substance use.
Kentucky	June–July 2020	6	3	5	Recruited from justice & service provider project partners	Impact of COVID-19 on usual practices, use of telehealth, unique challenges, needed adaptations, & and successes in serving justice-involved women with a history of opioid use.

### Analysis

All sites conducted independent analyses of their own interview transcripts. The analysis process had both inductive and deductive components. Illinois and Kentucky had respectively conducted initial waves of line-by-line analysis that resulted in a list of initial codes individually using qualitative data analysis software to organize their data—Illinois = MAXQDA 20.1 (Verbi Software, n.d.); Kentucky = Atlas.ti V8 (Scientific Software Development, 1997). The first step in the multi-site analysis for was all sites to review each other’s data collection tools to ensure overlap in inquiry that would be necessary to make the collaboration worthwhile (see Table 1 for overview of topics covered in interviews). Next, Illinois and Kentucky exchanged their list of preliminary codes to identify potential areas of overlap, discussing codes that seemed to be similar based on preliminary operational definitions. They then merged the agreed upon list of similar codes into a single code list. Each site then used the combined list in a deductive manner, applying it to guide a final round of analysis on their own transcripts (this was the first time any codes had been applied to Indiana's dataset, as they had not done any prior inductive coding). Coded segments were then merged into a single Excel spreadsheet for the final analysis, which involved identifying broader themes and synthesizing findings among the sites. Because the nature of the data prevented the addition of new cases, saturation was established at the point at which iterating between the identified themes and individual site data no longer yielded any new insights (Eisenhardt, 1989; Saunders et al., 2018).

## Results

Three overarching themes and four sub-themes were identified. Table 2 provides an overview of the different nuances of the themes as they relate to each site.

**Table 2:** Cross site comparison of specific details related pertaining to each identified themes

	How theme was observed at each site		
Theme	Illinois	Indiana	Kentucky
1. Criminal justice response and impact			
1a. Releases, arrests, & census impact	- Accommodate social distancing through reduced census by limiting arrests, releasing minor offenders, & placing on community supervision	-Accommodate social distancing through reduced census by not processing juvenile offenders as arrests	-Accommodate social distancing through reduced census by limiting arrests, releasing minor offenders, & placing on community supervision -Stop transfers between facilities to help pandemic procedure implementation
1b. Impact on pre-release planning & linkage to services	-Rapid release prevents appropriate community MOUD linkage -COVID-19 info prioritized over MOUD education -Some who were COVID-19 positive released before obtaining results	*-*Rapid release prevents staff from identifying youth with SUD	-Rapid release hinders community service linkage -Limiting visitors for social distancing prevents involving family & friends in treatment & recovery planning -Difficulties addressing COVID-19 concerns of those being released
2. Community providers treatment referral & intake changes	-Limiting of new intakes -Quarantine requirements make placements difficult	-Limiting of new intakes, with some providers completely stopping intakes	-Limiting of new intakes -Some stopped taking jail referrals because high jail COVID-19 rates -Lower census implemented to facilitate social distancing -Length of some residential treatment expanded
3. Implementation of telehealth & telejustice			
3a. Telehealth	-Treatment system largely benefited from telehealth implementation -Client access to necessary technology noted as barrier	-Treatment system largely benefited from telehealth implementation -Client access to necessary technology noted as barrier -Drawbacks when working with high-risk situations	-Treatment system largely benefited from telehealth implementation -Client access to necessary technology noted as barrier -Older clients not always as comfortable with telehealth
3b. Telejustice	-Telejustice implemented for court hearings	-Telejustice implemented for justice system intakes, court hearings	-Telejustice implemented for court hearings, community supervision - Telejustice noted as a benefit for clients reporting on supervision

### Criminal justice response to COVID-19 and impact

#### Releases, arrests, and census impact

Across all hubs, data demonstrated that jails and youth juvenile justice centers made attempts to lower the census within their walls to accommodate social distancing. This was accomplished in several ways: by releasing individuals with low-level or minor offenses, by placing individuals on supervision within the community, or (in the juvenile justice system) by not processing individuals as an arrest or bringing them to the intake center.

One Illinois jail staff interview participant described the decarceration process as a “mass increase of individuals leaving custody” from releases to the community, to supervision, or to electronic monitoring. One Kentucky jail staff participant estimated that their jail had experienced nearly a 60% population reduction at the time of their interview. New arrests were limited across all three sites, and jail staff in Kentucky discussed stopping transfers between facilities to help with implementation of pandemic-related measures, “We did no transporting of inmates out for a long time … as far as that goes, it did drop the numbers of inmates at each facility”.

Relatedly, Indiana's justice systems stopped arresting and detaining youth, as one intake officer in an Indiana rural county explained: “Anyone who commits a misdemeanor or status offense no longer comes to the intake center.” Even further, one judge explained how changes in arrests specifically impacted cases related to substance use: “the [referrals] we get are more of the violent-type crime referrals instead of substance abuse issues”. Regarding detained youth or those in placement, one justice system staff person explained they were considering what to do with youth on a case-by-case basis to reduce numbers for social distancing:

We immediately looked at any kids who were in placement to determine if we could change that placement [and] return them to a safe home environment. We also looked at kids in custody, to see if there’s any way we could release any of those kids, basically back to the community.

#### Impact on pre-release planning and linkage to services

While the quick release of individuals with low-level offenses was a critical protective measure to reduce COVID-19 spread, it limited jail staff members’ ability to support individuals in planning for release. According to one Illinois jail staff participant, the speed at which the releases happened meant many individuals who had or were scheduled to start MOUD “didn’t have time to be set up for an aftercare facility”. Additionally, the same facility had to divert time at discharge usually reserved for face-to-face MOUD treatment discussions to instead provide COVID-19 information: “…at that point in our release procedures previously, we would have taken time if somebody was interested to make sure they had everything they needed in terms of [MOUD], so we had to switch to… just providing packets of information”. Indeed, releases in this facility were happening so quickly some individuals who were tested for COVID-19 upon entry to the jail, and later were determined positive for COVID-19, were released before receiving their results. Similarly, in juvenile justice systems in Indiana, limiting justice system referrals, arrests, and intakes affected staff members’ ability to identify youth in need and connect them to SUD services. One probation officer explained concern over “not doing anything right now to get kids [with OUD] connected to services”.

Like Illinois and Indiana, correctional staff in Kentucky also discussed difficulties connecting incarcerated individuals with OUD to appropriate services. However, these interviews also pointed to the impact of limiting contact with family and friends during incarceration, particularly on recovery or treatment for OUD, and the need for flexible approaches:

We've been working hard on getting the family involved in the recovery…until COVID hit. But, I think we do need to be more mindful and creative on how we can…talk to the families of our clients, whether it be about Narcan [a brand name version of the opioid overdose reversing medication naloxone], getting it more accessible for our moms and dads out there that have sons and daughters that are actively using, that they can't get into treatment for whatever reason right now.

Kentucky respondents from jails also discussed how they were doing their best to address concerns from individuals preparing for release, especially those who were anxious about COVID-19 prevalence in the communities to which they were returning: “We again did our best to prepare [the individual]. We printed off all kinds of information…gave him a mask when [he] left, just to kind of prepare him as much as possible”.

### Community-based treatment providers: referral and intake changes

Data from all three states demonstrated considerable intake restrictions or limitations on the part of community-based treatment providers, particularly as related to justice-involved individuals. In Indiana, juvenile justice system staff expressed concern that they were unable to connect youth to needed services; one justice system worker explained how some outpatient substance use programs “just stopped” and were not providing any services. Similarly, two Illinois treatment providers discussed how they stopped taking referrals from the criminal justice system “because the jails were the highest concentration of COVID positive clients, and they were not testing”. This provider further explained that there was concern over placing clients and staff at risk of transmission or infection by accepting criminal justice referrals given high rates of COVID-19 in jails.

In Kentucky, general restrictions on new program intakes combined with quarantine requirements made it “quite challenging”, according to one respondent, to get newly-released individuals into treatment or recovery-oriented housing. One Kentucky representative also discussed how some individuals who were initially recommended to complete residential treatment were instead transferred to a less intensive level of care to minimize COVID-19-related risks:

…there were some clients that were just in residential programs, that they’re not controlled by DOC [the Department of Corrections], but we have a contract with them…and the ones that it was felt like they could go home and do…intensive outpatient [treatment] at home, they were able to send home. You know, just because – because we’re getting complaints from people who were in the halfway houses, or in the long-term residential programs, that ‘there are too many people here, we can’t properly social distance right’.

Illinois faced similar challenges; for instance, one treatment provider discussed how they had to cut capacity to ensure social distancing: “The biggest for us on the residential side is we went to a half census…that allowed us to do single clients to a room, so that they didn’t have to share space and/or share bathrooms”. Another provider discussed how a COVID-19 outbreak forced them to implement protocols to “isolate in place”, which forced them to stop taking new clients. This same provider also discussed how they extended the length of residential treatment from 90 days to 6 months because they recognized a need to provide more time for clients to find transitional or permanent housing options due to the pandemic. Although the longer duration of treatment services may have been a beneficial adaptation for many clients, it nonetheless highlights how housing challenges at re-entry were exacerbated during the height of the pandemic.

### Implementation of telehealth and Telejustice

#### Telehealth and SUD/OUD treatment

In response to increased rates of release from carceral settings and limited intakes to community treatment providers, participants from all three states described the implementation of telehealth services for SUD/OUD treatment. A Kentucky treatment provider discussed the benefits of the move to telehealth services for those in the community:

…that's [telehealth is] the way that we're doing things. I think some people like it. ... It's been a lot better for those with transportation issues…And now they're at home and they're showing up better. I think the state was good to respond as quickly as they did about providing telehealth…I think the response that happened from the state and federal level was good.

While most telehealth discussions were positive, some providers voiced concern. An Indiana provider explained their concern over telehealth for higher-risk youth: “not holding as much face-to-face contact creates some level of anxiety when you are talking about those types of high-risk situations”. Providers in all three states spoke of barriers related to phone access, limited data plans, and internet access that could impact client’s ability to engage in video-based services. In Kentucky, one provider also pointed to age as a potential barrier to telehealth engagement: “I think there is a comfort level for the age differences…I see the younger generation really are embracing it [telehealth] because that’s what they know and what they’re used to. But there’s a few that are a bit older that it’s been a struggle for”.

#### Telejustice

Data from all three hubs also described how the justice system adapted to providing virtual or telejustice services across different points of the system, including at intake, when interfacing with courts or treatment, and during contacts while on community supervision.

In Indiana, juvenile justice systems not only implemented a telejustice model for court hearings but also for youth coming in for justice system intakes following an arrest or system referral. One Indiana intake officer explained how telejustice for system intakes was made possible through collaboration between law enforcement and the juvenile justice system: “instead of just automatically putting [bringing to intake center] kids that they have encountered for delinquent activity or arrest…they consult with intake to see [if they can] go home [to conduct the intake remotely or virtually] or bring to the intake center”.

Kentucky's data described the utilization of telejustice services for individuals who were on community supervision. One Kentucky representative described how useful this approach was:

I think it’s [virtual reporting] been fantastic because you have people [on supervision] who are at work…They’re able to stay right there at work and report in….I think it’s helped them a lot. Just from, just a reporting aspect, it’s just easier for them. I mean, to be honest with you, it’s almost like, if me and you are in the same room as opposed to me and you talking here, what’s the difference?

In Illinois, telejustice was also used for drug courts. One treatment provider highlighted how telejustice facilitated this collaboration between them and the court system, “…we have a large portion of clients who are here [in treatment] as a mandate of their probation. So, we have moved from in-person visits [with the court] to telehealth [i.e., telejustice] visits”. This same provider discussed how this move was easy because they already had a pre-pandemic telejustice relationship with a drug court.

## Discussion

The current study highlights the impact of COVID-19 on the justice system and SUD treatment systems as reflected in data collected in three separate states. Most of the prior published literature in this area has focused on the treatment of SUD/OUD within jail and prison walls during the pandemic (Donelan et al., 2021), whereas our findings highlight issues existing at the boundaries between jails and treatment systems, how they have been impacted or changed by the pandemic, and their potential impacts on the lives of people with SUD/OUD. This presents an opportunity to identify potential actions that can be taken to improve such transitions in general and in preparation for future public health crises.

Data from all three hubs/sites described decarceration, early releases, or changes to pre-trial detentions (in the juvenile justice system) as safety measures taken in response to COVID-19. Such trends are consistent with reports from justice systems across the country (Kang-Brown et al., 2021; Minton et al., 2021), although some argue such measures are inadequate and further depopulation is necessary (Franco-Paredes et al., 2021; Kang-Brown et al., 2021). Further discussion and research surrounding decarceration and changes to other policies – such as pretrial detention regulations – are necessary, especially as recent evidence has highlighted that decarceration is a population-health benefit and an important anticontagion strategy (Reinhart & Chen, 2021). Despite this, there is evidence to demonstrate pandemic-based releases are linked to COVID-19 outbreaks in some communities (Reinhart & Chen, 2021), and stronger testing and discharge education could help mitigate such events. However, any COVID-19 education should be balanced to ensure individuals are also receiving appropriate information regarding available treatment and supports.

Across all three datasets, issues at the intersection of the criminal justice system and treatment systems were identified. While releases and decarceration are broadly seen as positive, this created issues with SUD/OUD community treatment linkage. This is because the combination of sudden decarceration and restrictions on community treatment intakes created a bottleneck with individuals not being consistently or expeditiously connected with services, a crucial issue given evidence for increased risk of and high rates of drug-related deaths following release from carceral settings (Jourdrey et al. 2019; Merrall et al., 2010; Victor et al., 2022). Service gaps created at the intersection of decarceration and reductions in available treatment services underscore the need to increase collaboration between the justice system and treatment providers.

Cross-system collaboration between the criminal justice system and service providers is a long-standing issue, especially with respect to substance use (VanderWaal et al., 2008). Issues such as differences in organizational structure and culture, competing philosophies (e.g., harm reduction vs. abstinence), as well as uncertainty in roles have historically hindered collaboration between systems (e.g., Kapp et al., 2013; Lasher & Stinson, 2020). Still, efforts have been made to improve collaboration between systems and research has identified multiple strategies for attaining this goal, such as through formal contracts, co-location of services, and information sharing (Fletcher et al., 2009; Lehman et al., 2009). As a result, many systems have developed successful collaborative relationships, and research has demonstrated such systems value collaboration (e.g., citation blinded; Guerrero et al., 2014; Lasher & Stinson, 2020). Further research is needed to explore the impact of COVID-19 on other cross-system relationships that may impact this population or other similar populations, such as those involved in the criminal justice system with severe mental illness.

The focus on justice-involved individuals with SUD/OUD also raises the question of decriminalization of drug use (Bonn et al., 2020; Kleinman & Morris, 2021; Maynard & Jozaghi, 2021). From population health lens, one solution to avoid the need for mass decarceration and associated problems in the event of another pandemic is to reduce the number of incarcerated individuals by decriminalizing certain activities such as drug use (del Pozo & Beletsky, 2020). However, some argue that decriminalizing drugs would only increase drug use and thus even further increase burden on the healthcare system (see Bonn et al., 2020 for discussion). It should also be noted that discussion of decriminalization of drug use has considerably different ramifications for the juvenile as compared to the adult justice system, and a larger focus on diversion to treatment for juveniles should be emphasized. Regardless, additional research examining the outcomes of decarceration, diversion, and reduced arrest rates in response to COVID-19 could offer useful insights into potential impacts of drug reform.

Finally, the expansion of telehealth may be one of the most defining adaptations to behavioral healthcare that has resulted from the pandemic (Becker et al., 2021; Hughto et al., 2021; Pierce et al., 2021). Positive views of telehealth identified in this study are generally consistent with those found in most of the behavioral health literature, and there has been a call by experts to ensure many of the changes that have improved telehealth access remain in place after the pandemic’s end (Bergman & Kelly, 2021; Davis & Samuels, 2021; Samuels et al., 2020). While discussions of telehealth’s benefits were often broad, retaining pandemic-based changes in telehealth along with changes in MOUD dosing regulations would have particular benefits for improving post-release agonist-based (e.g., methadone or buprenorphine) MOUD treatment linkage and retention (Davis & Samuels, 2021). However, other research has noted concerns regarding telehealth among clinicians, such as increased diversion of medications for addiction treatment and potential increased overdose risk (Hunter et al., 2021). Despite flexibility these relaxed regulations provide, individuals might still have difficulties attending the minimal number of office visits (e.g., screening of vitals, picking up methadone doses) that could be required of their providers. In these cases, the use of long-acting injectable naltrexone or buprenorphine provide additional options that only require a single monthly office visit for medication administration. Findings from all three hubs/sites in the present study also highlighted barriers to conducting telehealth and telejustice, such as phone and internet access, which made it difficult to connect to some populations. Thus, results highlight the importance of continuing to implement hybrid models of care to best meet individuals’ needs and circumstances as the US emerges from the peak of the pandemic.

### Limitations

The primary limitation of the study is the use of data collected by different teams whose work was in different locations, focused on different populations of justice-involved people with SUD, and using different qualitative approaches and data collection instruments. However, the common themes identified highlight the value of synthesizing data across multiple projects that reflect different but related state-embedded systems. In particular, the use of rapid qualitative approaches in light of the need to document ephemeral phenomena reflective of the pandemic that would be difficult to capture retrospectively limited their sample sizes and depth of questioning. Given this, the ability to identify common themes underscores the salience of the issues identified by justice systems and treatment providers. This consistency is all the more notable given the diversity of stakeholders included as study participants, including administrative or supervisory corrections staff (Kentucky), juvenile intake and probation officers (Indiana), front-line jail staff (Illinois and Kentucky), and community-based treatment providers offering diverse services along the continuum of care (Illinois, Indiana, and Kentucky). Examined individually, each state or system’s findings would have been relevant and timely; combined, they speak both to the impact of COVID-19 across diverse systems (particularly those that affect vulnerable, justice-involved populations), as well as COVID-19’s impact on inter-systemic partnerships, including those required for effective transitions between carceral and treatment settings.

Two other limitations include the lack of information related to specific types of MOUD and the continuums of care within which each research hub’s work was set. In the case of the former issue, there was not enough information to develop strong themes related to specific MOUDs versus general issues with treatment linkage due to: the relatively small sample sizes, differences in specific data collection instruments, and the fact that Indiana‘s data did not lend to this given prescribing MOUD to youth is not common within their system. Regarding the latter issue, the structure of MOUD care and post-release linkage is related more to the individual carceral setting and available community services (Scott, Dennis, Grella, Mischel, et al., 2021; Scott et al., 2022), making it difficult to compare how themes impacted general continuums of care across states.

## Conclusions

The current study offers important insight into the impact of COVID-19 on individuals with OUD who are involved in the justice system. Results highlight that rapid adaptations made by both the criminal justice and treatment systems to maximize the safety of individuals likely resulted in unintended consequences hindering treatment access. Most glaring is the potential bottleneck created when scores of people with SUD/OUD were released to communities when treatment providers were limiting new intakes. Research and policy work is still needed to improve outcomes for this vulnerable population; however, findings regarding systems’ ability to adapt to the pandemic show promise.

## Data Availability

Qualitative data are not available due to confidentiality concerns related to such a small sample.

## Abbreviations

COVID-19: Coronavirus 2019
JCOIN: Justice Community Opioid Innovation Network
OUD: Opioid use disorder
MOUD: Medications for opioid use disorder
SUD: Substance use disorder
